# Mind it! A mindfulness-based group psychotherapy for substance use disorders in adolescent inpatients

**DOI:** 10.1007/s00787-024-02465-z

**Published:** 2024-05-15

**Authors:** Tanja Legenbauer, Christiane Baldus, Carina Jörke, Lara Kaffke, Amra Pepic, Anne Daubmann, Antonia Zapf, Martin Holtmann, Nicolas Arnaud, Rainer Thomasius, the IMAC-Mind Consortium

**Affiliations:** 1 Landschaftsverband Westfalen-Lippe (LWL) University Hospital Hamm for Child and Adolescent Psychiatry, Ruhr-University Bochum, Heithofer Allee 64, 59071 Hamm, Germany; 2 German Center for Addiction Research in Childhood and Adolescence, University Medical Center Hamburg-Eppendorf, Hamburg, Germany; 3 Institute of Medical Biometry and Epidemiology, University Medical Center Hamburg-Eppendorf, Hamburg, Germany

**Keywords:** Substance use disorders, Adolescents, Cannabis, Mindfulness, Treatment

## Abstract

Cannabis use disorder (CUD) is the most frequent reason for psychiatric inpatient substance use disorder (SUD) treatment among 15–19-year-olds in Germany. Despite effective treatment programs, relapse rates remain high. Thus, existing multi-component programs (TAU) need to be enhanced with SUD-specific elements. Mindfulness-based interventions (MBI) seem promising as they can positively influence SUD-related behaviors (e. g. craving). Given limited research in adolescents, this randomized controlled trial investigated the extent to which MBI-based group therapy (Mind it!) as an add-on treatment to TAU led to fewer cannabis use days after 6 months in 84 adolescent inpatients with CUD. Additionally, craving, severity of CUD, and changes in mindfulness were monitored (pre-, post-, and follow-up (FU) assessments). The results revealed a significant reduction in cannabis use days in both groups at 6-month FU (d = − 0.72 and = − 0.75). Although minor additional benefits of Mind it! were evident post-treatment, specifically reduction of craving and SUD severity, by the 6-month mark, TAU exhibited a more substantial decrease in SUD severity (d = 0.78), and reward craving (d = 0.28) compared to Mind it!. Regarding self-regulation skills (mindfulness), Mind it! demonstrated superiority over TAU after 6-month FU (d = 0.27). Therapists judged the MBI as feasible. (Serious) adverse events were unrelated to Mind it!. There was a systematic dropout among Mind it! participants. Primarily, the results emphasize the effectiveness of TAU in reducing cannabis use. MBI also seem feasible for youth, but results remain inconsistent and unstable over time. Importantly, enhanced adherence to reduce dropouts is needed.

**Trial registration:** German Clinical Trials Register, DRKS00014041. Registered on 17 April 2018.

**Supplementary Information:**

The online version contains supplementary material available at 10.1007/s00787-024-02465-z.

## Introduction

The use of alcohol and other drugs has a significant negative impact on health outcomes. Around 35 million people worldwide suffer from a substance use disorders (SUD) and 585,000 people died in 2017 as a result of substance use [1]. In total 19.737 of Disability adjusted life years (DALYs) were attributed to drug and alcohol use outcomes [2]; thereby, according to the World Health Organisation (WHO) more than 9% of DALYs due to psychoactive substance use were caused in people under the age of 24 [3]. While the majority of adolescents report temporary drug use, a minority transition from early, potentially harmful use to chronic substance use disorder (SUD; [4, 5]). Internationally, approximately 11% of youth meet criteria for any SUD [6]. According to a large German survey, alcohol use disorder (10.1%) and cannabis use disorder (CUD, 2.6%) are the most common SUDs among adolescents (12–18 years; 12-month prevalence; [7]). Cannabis use and the need for CUD treatment have increased worldwide in the last decade [8], and in Germany, CUD is the most common reason for psychiatric inpatient SUD-related treatment in adolescents [9]. These findings underscore two critical points: (1) adolescence is a crucial period for establishing problematic substance use, and (2) SUD, particularly CUD, is a significant health risk for young populations. Despite effective treatments for youth with SUD [10], the risk of relapse is high, especially in the first year post-treatment [9]. To reduce relapse rates and prevent chronic substance use, it is essential to address maladaptive substance use patterns in their early stages, necessitating tailored treatment elements that target SUD-specific maintenance mechanisms. Research has highlighted the significance of dysregulated neural processes, particularly in reward learning and executive functioning, in the development and maintenance of SUD [11]. Namely self-regulation and related affective reactivity as part of executive functioning seem to play critical roles in the development and maintenance of SUD [11]. However, these findings have not been well incorporated into treatment approaches, particularly for adolescents, where research is limited [10]. Mindfulness-based approaches represent a promising solution, as they address key elements associated with SUD-related behaviors e.g. self-regulation skills, impulsivity and motivational aspects that underly SUD [12–15]. Mindfulness fosters a non-judgmental awareness of the present moment, helping to stabilize cognitive and emotional functioning (e.g. [16]), improve coping mechanisms (e.g. [17]), and reduce stress-related cravings [18]. e.g. by bringing the attention to automated and uncontrolled habits that are linked to substance craving and use, these behaviours can be modified [19, 20].

Various mindfulness-based interventions (MBIs) have been developed and adapted for use in SUDs over the last three decades. For example, Mindfulness-Based Relapse Prevention (MBRP) integrates mindfulness-based practice and behavioral-based relapse prevention techniques [21]. The approach has been tested in adult clinical populations indicating—even compared to active control groups—that e.g. days of substance use and heavy drinking could be reduced with small clinical effects up to 6 months follow-up. Recent meta-analyses in adult samples yielded promising evidence [22] suggesting that 1. MBIs reduce craving levels better than other active programs such as 12 steps focused programs or cognitive behavioral therapies (n = 3541 individuals from 37 studies) and 2. the amount of days with substance use decreased slightly after MBIs compared to other established treatments such as relapse prevention at post and follow-up measurement (n = 2825 participants from 35 studies [23]). These meta analyses also indicated a high risk of bias due to high heterogeneity of studies relating to heterogenous samples (e.g. both inpatient and outpatient settings), various MBI treatment approaches [e.g. MBRP, mindfulness-based stress reduction, but also acceptance and commitment therapy (ACT) approaches] and various kinds of substance dependencies (e.g. alcohol, cannabis, other illicit drugs). In addition, there are few studies focusing on minors with SUD or substance use problems.

A meta-analysis in children and adolescents receiving MBIs, not including adolescents with SUD, showed inconsistent findings, and only small effects regarding stress/anxiety reduction or an increase in mindfulness [24]. There are only some preliminary studies in adolescents with SUD. In an uncontrolled pilot trial [25], Himelstein et al. reported positive effects on healthy self-regulation (an aspect of mindfulness) in incarcerated youth, a group with notable substance use issues. However, changes in substance use were not investigated. Another study in adolescent outpatients with SUD (n = 54; age 14–18 years), found a reduction in alcohol use (but not cannabis) when participants were randomized to a wait list control or a four-week group treatment focusing on urge surfing, a mindfulness-based technique for overcoming substance cravings [26]. In contrast, a small sample of adolescent outpatients (N = 37, aged 13.7–20.4 years) with comorbid posttraumatic stress disorder and SUD showed a reduction in cannabis use, but no changes in alcohol or nicotine use, following an MBI [27]. Moreover, evidence in adolescents suggests an inverse association between mindfulness and craving (e.g. [28]).

In sum, MBIs aim to address the underlying mechanisms of SUD by enhancing self-regulation skills. While the application of MBIs in youth with SUD seems promising, the evidence remains limited due to small sample sizes, design limitations, mixed age groups, and setting-related issues. This study seeks to contribute to this emerging field by examining the additional benefits of a SUD-specific group MBI (Mind it!) for adolescent inpatients with a particular focus on cannabis use, a common reason for adolescent inpatient SUD treatment in Germany [29]. We assumed that Mind it! would lead to a lower number and greater reduction of days with substance use regarding (1) cannabis (assessed with the time line follow back interview, primary outcome), (2) alcohol, and (3) overall substance use within the last 30 days, 6 months post-intervention. Additionally, we assumed direct intervention effects (pre-post assessments) on craving, SUD severity, and mindfulness for Mind it! participants compared to treatment as usual (TAU). Finally, we assessed the feasibility and acceptability of the intervention.

## Methods

### Design and procedure

The study was originally designed as a confirmatory randomized controlled trial (RCT; see [30]). However, because of enrollment difficulties resulting from early (disciplinary) discharge, initiation of rehabilitation or cessation of treatment after qualified withdrawal (see also [31]), alongside challenges in maintaining participation during the study period (such as early discharge, scheduling conflicts, and motivation issues, it was transformed into an exploratory study with two conditions, focusing on the feasibility of Mind it! in adolescent inpatients with SUD. To convert the study after one year of data collection into an exploratory one, we submitted an appropriate application to the sponsor where we laid out the reasons for problems in recruitment and all measurements that have been taken so far and presented a new power analysis for conducting a feasibility study. Participants were recruited from two inpatient university hospitals with specialized wards for SUD treatment in two German cities. Eligibility criteria included an ICD-10 SUD diagnosis (F10–F19), age between 13 and 19 years, and intention to receive further psychiatric inpatient SUD treatment after qualified withdrawal. Exclusion criteria encompassed not meeting SUD criteria, no substance use in the 30 days before treatment, acute psychotic or suicidal symptoms, flashbacks, and limited language or intellectual capacity. Both centers provided TAU and the MBI alongside TAU (Mind it!). During the survey period, our study nurse screened all newly admitted inpatients to qualified withdrawal in our electronical hospital information system for potential inclusion based on predefined inclusion and exclusion criteria. If a patient met the inclusion criteria, the therapist and nursing team were notified, and the patient, along with their legal guardians, was briefed about the study by a study staff member. If patient and legal guardians were willing to participate, informed consent was obtained. Upon receipt of consent, the patient was eligible for inclusion in the study and randomized after completing withdrawal treatment successfully. Two baseline appointments (t0a, t0b) were conducted, including structured interviews and clinical assessments. Diagnoses were based on clinical records and structured interviews by trained study psychologists. Disagreements between record and interview-based diagnoses were intensively discussed. Eligible patients were randomized to either TAU or Mind it! (1:1 allocation ratio, stratified by center with variable block length). Research assistants conducting the post-treatment assessments were blinded to group allocation. Diagnostic assessments relevant for the present analyses occurred at 8 weeks (t2), and 6 months post-intervention (t3). The study protocol was published [30] and the trial was registered at the German Clinical Trials Register, DRKS00014041, where modifications to the study design were also registered. For changes to the study protocol see also information in supplemental material.

### Participants

**Fig. 1:**
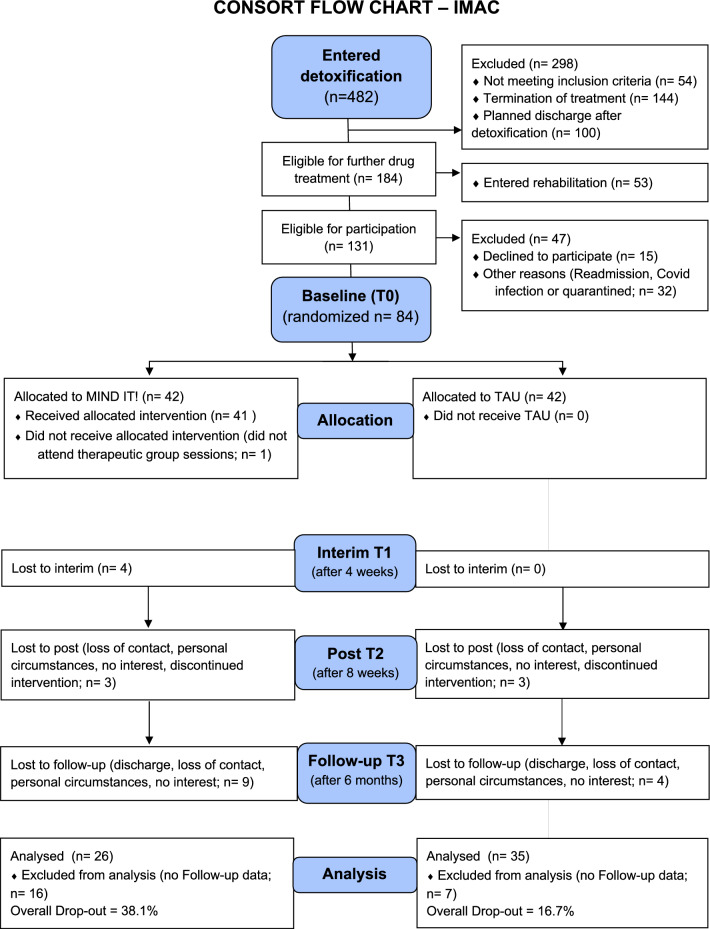
Patients flow. Patient flow from admission to detoxification treatment to follow-up is displayed. Mind it! = add-on mindfulness treatment group over 8 weeks; TAU = treatment as usual. One patient in the Mind it! group did not participate in any of the group sessions due to early discharge; as data were available for T0 and T2, this patient is part of the ITT population

Recruitment occurred from July 2018 to October 2020 at both study sites. Upon enrolment, participants had all completed phase 1 (withdrawal treatment) and were receiving specialized adolescent psychiatric SUD inpatient treatment. Of 482 patients who entered qualified withdrawal during the recruitment period, only 131 met the study's eligibility criteria, with 47 patients excluded before randomization (see Fig. 1). These exclusions were attributed to various reasons: refusal to participate, multiple prior admissions during the study period and previous participation, or COVID-related illness and subsequent quarantine, rendering them unsuitable for study inclusion. Thus, 84 patients (intention-to-treat, ITT population) were randomized, with 42 assigned to the Mind it! group and 42 to the control group. Of these 84 participants, 61 provided complete data sets (retention rate at follow-up: 72.6%, details see Fig. 1).

The original power analysis, as described in Baldus et al. [30], indicated a required sample size of 123 participants per group to detect treatment effects, assuming small to medium effects in reducing substance use days after 6 months. However, we aimed to determine the sample size needed to reliably assess the intervention's feasibility and initial effectiveness. This analysis revealed that 85 patients (43 in the intervention group and 42 in the control group) would be sufficient to demonstrate a standard mean difference (Cohen's d) of 0.61 in the primary outcome (Timeline Followback (TLFB) cannabis use days), with a power of 80% and a 5% type-1 error rate for a two-sided hypothesis. Smaller effects may not be detected with the reduced sample of 85 patients.

### Treatment

#### Standard SUD treatment (TAU)

Treatment for adolescent SUD patients at the two study sites is routinely conducted in an inpatient approach [32] and divided into two therapy phases: The first primarily comprises medical and psychiatric supportive *qualified withdrawal* (phase 1) in a protected setting. Within this framework, a detailed psychiatric, neuropsychological, and psychosocial diagnostic procedure is performed. Furthermore, psychoeducational interventions are conducted to understand the individual background of drug use and to strengthen motivation to continue treatment after phase 1. The subsequent *post-withdrawal treatment* (phase 2) focuses on comorbid psychiatric problems associated with the individual problematic substance use. Through psychoeducation, patients are supported to motivate themselves intrinsically for an abstinent life and develop skills for relapse prevention. This multimodal treatment concept mainly consists of psychotherapeutic, somatic, and medical interventions, complemented by kinesiotherapy, family therapy, occupational and educational therapy, and music therapy. The described approach reflects the standard procedures of SUD treatment according to the national treatment recommendations in Germany [9, 32, 33].

#### Mind it!

The MBI Mind it! is an add-on to TAU in the above-described inpatient setting. The intervention manual was translated and adapted to the inpatient setting (for details see [30, 31]) based on the manual by Himelstein et al. [34]. Elements in Mind It! particularly rely on exercises already present in MBRP and its adaptation to SUD treatment by Himelstein, encompassing psychoeducation, a variety of meditation exercises including compassion meditations, guidance for daily independent meditation practice, mindfulness-based strategies for managing cravings, informal mindfulness exercises for addressing impulsivity, practices for observing emotions mindfully, and exploration of substance use's impact on interpersonal relationships. The development of Mind It! adhered to established principles for adapting mindfulness-based interventions for adolescent participants, incorporating shortened session and meditation durations, increased session frequency, and age-appropriate language adjustments [35] alongside an interactive group session design. Initially, meditation sessions were limited to 5–10 min and gradually extended across subsequent sessions. The manual provided detailed guidance, outlining procedures and offering example formulations for each session component (e.g., psychoeducational content, practice instructions, and post-session inquiry). Additional information regarding session details and content can be found in supplementary materials. During the newly established *4-week core phase* with two sessions per week, a basic understanding of mindfulness and a training routine for formal meditation is established. Sessions commenced with a welcome and mindful check-in, followed by a discussion on independent practice and session-specific content. Tasks were explained at the end of each session, including an encouragement to engage in formal mindfulness exercises outside of group sessions. The following 4 weeks are used to deepen the formal training with more intense meditation once per week. Treatment fidelity was ensured by intensive training before and during the intervention period. The training included three 2–3-day workshops with an experienced expert in mindfulness-based therapy to learn and discuss MBI methods such as attitude, own practice experiences, and inquiry techniques. To ensure adherence to the mindfulness concept and rationale, regular supervision by the same expert was provided. Results regarding the acceptability of the manual by patients has been published elsewhere [31]. The study results indicated that participants of the Mind it! group “perceived confidence” (M = 3.43, SD = 1.01; range 0–6), and experienced “own inner involvement” (M = 4.00, SD = 0.91; range 0–6); however, some patients reported that some exercises were perceived less positively by patients. For example, some formal meditation exercises were difficult to endure.

### Measurements

Since the study’s objective shifted away from a fully powered trial (primary outcome: substance use at follow-up) to an exploratory trial, the primary outcome (cannabis use days) was assessed using the Timeline Followback (TLFB) interview [36], and secondary outcomes were limited to (a) use days of alcohol and overall substance use, SUD severity (Severity of Dependence Scale, SDS,^1^ [37]), craving (Cannabis Craving Screening CCS-7^2^; [38, 39]), and participants’ mindfulness (Healthy Self-Regulation subscale from the Mindful Thinking and Action Scale for Adolescents, MTASA, and the Mindful Attention Awareness Scale for Adolescents (MAAS-A; [40]). Furthermore, to estimate feasibility, the Structured Assessment of Feasibility tool (SAFE, [41]^3^) was applied and the number of adverse events (AEs) and serious adverse events (SAEs) during the treatment period were recorded. Detailed descriptions of the instruments are provided elsewhere [30].

### Statistical analysis

All analyses were performed using SPSS 26.0 for Windows. The primary outcome (number of days with cannabis use at t3) was analyzed as change from baseline using a linear model with treatment group and recruitment center as fixed effects and number of days with cannabis use within the past 30 days at baseline as a covariate. We report model-based group differences (adjusted for baseline and center) with corresponding 95% confidence intervals and p-values. The secondary outcomes (alcohol use days, overall substance use days) were analyzed applying the same model approach as in the primary analysis. The further secondary outcomes (mindfulness, craving, SUD severity) were evaluated using the same model approach extended to a mixed model with the same covariate and fixed effects as in the primary analysis and time as additional fixed effect and patient as a random effect to account for repeated measurements at t2 and t3. Mixed models provide unbiased estimation if the missing at random assumption holds. Additionally, we performed sensitivity analyses by imputing missing values using multiple imputation [43]; details on the statistical procedure and the results of these sensitivity analyses are reported in the supplementary information (SI) if not significantly different from those reported here. Safety endpoints (AEs and SAEs as well as SAFE scores) were compared between the treatment groups according to their distribution, i.e. using analysis of variance tests and Chi^2^ tests to compare frequencies. We report Cohen’s d as a measure of effect size (Cohen's d, standardized mean difference: small (0.20), moderate (0.50), large (0.80) [44]). The significance level was set at 0.05.

## Results

### Baseline characteristics

The two groups were similar in terms of age, sex, educational level, migration background, and comorbidity as well as SUD severity, craving, and mindfulness levels at baseline. There were no differences in type of drug or number of use days. The sample exhibits significant illness severity, with an average of five comorbid diagnoses per participant. The majority of participants experience comorbid affective or externalizing disorders. Although there seems to be lower anxiety levels in the TAU group compared to the MBI group, this difference did not reach statistical significance [Chi^2^ (84,1) = 3.614, p = 0.057]. Additionally, PTSD and borderline personality disorder are present in approximately one-fifth of the participants. The average duration of treatment in the post-acute psychiatric inpatient setting was approximately 49 days. On average, Mind it! participants attended 49.8% (5.98) of the 12 mindfulness sessions (range 1–12). Baseline characteristics of enrolled patients are detailed in Table 1.

**Table 1:** Baseline characteristics for full sample and by group

Variable	(n)	Total	Mind it! group	Control group
Demographics				
Age [years; M (SD)]	84	16.38 (1.22)	16.33 (1.16)	16.43 (1.29)
Sex [male; % (n)]	84	63.1% (53)	54.8% (23)	71.4% (30)
Migration background [German; % (n)]	64	90.6% (58)	93.8% (30)	87.5% (28)
Current school attendance [yes; % (n)]	65	69.23% (45)	65.6% (21)	72.7% (24)
No. of comorbid diagnoses [M, (SD)]	84	4.98 (2.07%)	4.83 (2.14)	5.12 (2.00)
Depression [%, (n)]	84	64.29% (54)	6.67% (28)	61.91% (26)
Anxiety disorders [%, (n)]	84	20.23% (17)	28.57% (12)	11.09% (5)
Externalizing disorders [%, (n)]	84	82.14% (69)	76.19% (32)	88.01%(37)
PTSD [%, (n)]	84	19.05% (16)	19.05% (8)	19.05% (8)
Borderline personality disorders [%, (n)]	84	17.86% (15)	19.05% (8)	16.67% (7)
Prevalence of use (TLFB)				
Cannabis [% (n)]	84	88.1% (74)	88.1% (37)	88.1% (37)
Alcohol [% (n)]	84	82.1% (69)	83.3% (35)	81.0% (34)
Cocaine (% (n)]	84	32.1% (27)	28.6% (12)	35.7% (15)
Amphetamine [% (n)]	84	46.4% (39)	42.9% (18)	50.0% (21)
Substance use days^a^ (TLFB)				
Cannabis [M (SD)]	74	20.6 (11.18)	19.86 (11.25)	21.35 (11.21)
Alcohol [M (SD)]	69	7.43 (8.49)	8.77 (10.15)	6.06 (6.22)
All substances [M (SD)]	84	28.43 (5.42)	28.6 (5.06)	28.26 (5.81)
SDS (M (SD)]	76	7.88 (2.64)	7.46 (2.46)	8.32 (2.78)
CCS-7				
Reward craving, (M (SD)]	70	3.80 (2.08)	3.83 (2.02)	3.78 (2.17)
Relief craving, (M (SD)]	70	3.14 (1.67)	3.21 (1.66)	3.08 (1.70)
Mindfulness (MTASA)				
Healthy self-regulation (M (SD)]	76	2.96 (0.70)	2.96 (0.78)	2.92 (0.61)

*(n)* case number, *SDS* Severity of Dependence Scale; *CCS-7* Cannabis Craving Screening short form, *MTASA* Mindful Thinking and Action Scale for Adolescents, *TLFB* Timeline Followback interview

^a^Last 30 days prior to admission. Prevalence of use within the last 30 days. M = mean, SD = standard deviation

Dropout analyses indicated that allocation to Mind it! was associated with dropout at follow-up. To examine dropout effects while considering various potential factors, we initially investigated the relationships between data availability at t3 and key participant characteristics. Detailed information can be found in the Supplement. We found significant associations, indicating a higher likelihood of data unavailability at t3 for participants in the intervention group (phi coefficient = − 0.24, p = 0.028), those with lower scores in mindfulness domains such as paying active attention (r = 0.27, p = 0.021) and being aware of observations (r = 0.32, p = 0.004), and individuals reporting fewer substance use days in the past 30 days (r = 0.22, p = 0.047). In an exploratory analysis, logistic regressions were utilized to forecast data availability at t3 based on previously significant variables. The final model revealed notable effects for group allocation (OR = 0.30, p = 0.044) and MTASA awareness of observations (OR = 3.58, p = 0.011), while the number of overall substance use days within the past 30 days showed no meaningful association (OR = 1.07, p = 0.121) [for further details see Supplementary Information, SI1)].

### Effects on substance use

Regarding the primary outcome (*cannabis use days in past 30 days),* the results showed no significant difference between the groups, but did reveal a relevant decrease in both groups, with moderate to large effect sizes. Regarding secondary outcomes, there was no relevant decrease in alcohol use days over time in the Mind it! group, but a reduction emerged in the TAU group (small effect size). The reduction in days of use (across all substances) was also small and did not reveal a relevant effect for either group; no relevant difference between the groups emerged. Mean values and test statistics are reported in Table 2 (for sensitivity analyses, see Table SI2.1).

**Table 2:** Results of linear model analyses for use days for the assessed substances at baseline and follow-up assessment

	Change from baseline								Between-group differences (CG–IG)			Effect size
	Mind it! group				Control group							
	EM	95% CI	p	d	EM	95% CI	p	d	EM	95% CI	p	d
TLFB												
Cannabis use days												
t0 to t3	− 8.98	− 13.90, − 4.07	< 0.001	− 0.72	− 9.26	− 13.42, − 5.11	< 0.001	− 0.75	− 0.28	− 6.70, 6.14	0.930	0.02
Alcohol use days												
t0 to t3	− 0.78	− 3.23, 1.68	0.528	− 0.12	− 2.12	− 4.20, − 0.05	0.045	− 0.35	− 1.34	− 4.54, 1.85	0.403	0.22
Overall use days												
t0 to t3	− 0.90	− 3.60, 1.81	0.510	− 0.13	− 1.67	− 3.96, 0.61	0.148	− 0.25	− 0.78	− 4.30, 2.74	0.540	0.11

t0 = baseline, t3 = follow-up

EM=Estimated Marginal Means for differences, negative target values represent a decrease in substance use between t0 and t3, positive values represent an increase in use. Effect size for intervention—a negative score indicates a decrease in number of use days within the past 30 days between baseline and follow-up, a negative effect size (Cohen’s d) is associated with a favorable intervention effect of the Mind it! group. CI=95% confidence interval; d: Cohen’s d; CG: control group; IG: Mind it! intervention group; t0: Baseline; t3: follow-up

### Direct intervention effects and follow-up results for SUD-related symptoms

Regarding *SUD severity (SDS),* no relevant differences between the groups emerged at post-assessment. At follow-up, however, there was a relevant difference between the two groups, with TAU patients showing a decrease on the SDS (d = 0.78). For *reward craving*, there was no relevant difference between the groups either for post-assessment or for follow-up changes; however, the effect size for group comparisons revealed a small effect in favor of Mind it! at post-assessment and in favor of TAU at follow-up. Regarding *relief craving,* there was a relevant difference between the groups insofar as Mind it! showed a stronger change with a large effect from pre- to post-assessment, whereas the change in TAU was negligible. However, this effect was not stable over time and was no longer detectable at the 6-month follow-up. Here, the groups did not differ regarding change in relief craving, showing small (Mind it!) to moderate (TAU) changes over time. An overview of the described results including mean values and test statistics as well as effect sizes is presented in Table 3 (sensitivity analyses Table SI2.2).

**Table 3:** Group differences and changes over time for SUD-related parameters at post-treatment and follow-up assessments

	Change from baseline								Between-group differences (IG—CG)			Effect size
	Mind it! Intervention group				TAU group							
	EM	95% CI	p	d^a^	EM	95% CI	p	D^a^	EM	95% CI	p	d^b^
SDS												
t0–t2	− 0.27	− 1.17, 0.64	0.560	− 0.11	0.24	− 0.63, 1.11	0.586	0.10	− 0.51	− 1.73, 0.72	0.416	− 0.20
t0–t3	0.54	− 0.58, 1.66	0.343	0.21	− 1.43	− 2.33, − 0.52	0.002	− 0.57	1.96	0.55, 3.38	0.007	0.78
CCS7												
Reward craving												
t0–t2	− 0.73	0.1.30, − 0.15	0.014	− 0.44	− 0.32	− 0.86, 0.23	0.249	− 0.19	− 0.41	− 1.20, 0.37	0.302	− 0.25
t0–t3	− 0.23	− 0.91, 0.46	0.517	− 0.14	− 0.69	− 1.25, − 0.14	0.015	− 0.42	0.47	− 0.41, 1.35	0.295	0.28
Relief craving												
t0–t2	− 0.98	− 1.43, − 0.53	< 0.001	− 0.76	− 0.14	− 0.56, 0.28	0.500	− 0.11	− 0.83	− 1.44, − 0.22	0.008	− 0.65
t0–t3	− 0.43	− 0.96, 0.10	0.107	− 0.35	− 0.64	− 1.07, − 0.21	0.004	− 0.50	0.21	− 0.47, 0.89	0.544	0.17

EM = estimated marginal means for differences, CI = 95% confidence interval; p = probality level for changes from baseline; effect size ‘d’: Cohen’s d; CG: control group; IG: Mind it! intervention group; t0: Baseline; t2: post-assessment; t3: follow-up. SDS = Severity of Dependence Scale; CCS-7 = Cannabis Craving Screening short form

^a^Negative d-score associated with favorable development over time

^b^Negative d-score associated with better results for Mind it! group vs. control group

### Enhancement of mindfulness due to Mind it!

The two groups showed similar, relevant improvements in ‘healthy self-regulation’ at post-treatment. At follow-up, the effect size indicated a relevant group difference, with Mind it! showing a moderate increase over time and a smaller effect for TAU. Both groups showed increases in mindfulness as measured by the MAAS, but no superiority of Mind it! over TAU was detected (see Table 4, sensitivity analyses Table SI2.3).

**Table 4:** Group differences and changes over time for mindfulness parameters at post-treatment and follow-up assessments

	Change from baseline								Between-group differences (IG—CG)			Effect size
	Mind it! group				Control group							
	EM	95% CI	p	d^a^	EM	95% CI	p	d^a^	EM	95% CI	p	d^b^
MTASA												
Healthy self-regulation												
t0–t2	0.19	0.01, 0.37	0.037	0.38	0.19	0.02, 0.35	0.025	0.37	0.01	− 0.24, 0.24	0.970	0.01
t0–t3	0.29	0.09, 0.49	0.005	0.59	0.15	− 0.01, 0.32	0.072	0.31	0.14	− 0.13, 0.40	0.307	0.27
MAAS												
t0–t2	4.85	0.86, 8.84	0.018	0.43	4.90	1.16, 8.64	0.011	0.43	− 0.05	− 5.37, 5.46	0.987	0.00
t0–t3	0.78	− 3.66, 5.23	0.728	0.07	2.78	− 1.02, 6.58	0.150	0.25	− 1.99	− 7.81, 3.82	0.498	− 0.18

t0 = baseline, t2 = post, t3 = follow-up

EM = Estimated Marginal Means for differences, CI = 95% confidence interval; p = probality level for changes from baseline; effect size ‘d’: Cohen’s d; CG: control group; IG: Mind it! intervention group; t0: Baseline; t2: post-assessment; t3: follow-up. MTASA = Mindful Thinking and Action scale for Adolescents; MAAS-A: Mindful Attention Awareness Scale for Adolescents.

^a^Positive d-score indicates better healthy self-regulation development over time

^b^Positive d-score associated with better results for Mind it! than control group

### Feasibility and safety

**Fig. 2:**
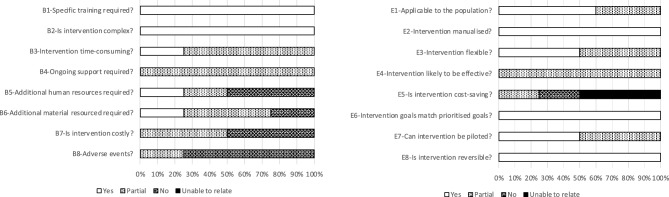
SAFE evaluation. Answers on the SAFE questionnaires as yes/partial/no or unable to rate in response to the following questions: 2a blockers: B1 “Does staff require specific training to deliver the intervention?”, B2 “Is the intervention complex?”, B3 “Is the intervention time-consuming to provide?”, B4 “Does the intervention include/require ongoing support and supervision?”, B5 “Does the intervention require additional human resources?”, B6 “Does the intervention require additional material resources?”, B7 “Is the intervention costly?” B8, “Are there any known serious or adverse events associated with the intervention?” and 2b enablers: E1 “Is the intervention applicable to the population of interest?”, E2 “Is the intervention manualised?” E3 “Is the intervention flexible?”, E4 “Is the intervention likely to be effective?”, E5 “Is the intervention cost-saving?”, E6 “Do the intended goals of the intervention match the prioritised goals?”, E7 “Can the intervention be piloted?”, E8 “Is the intervention reversible?”

The SAFE results provided partial support for the feasibility of Mind it!. The majority of the therapists evaluated the group program as suitable for young SUD patients (E1). The program was manualized (E2) and shows potential for flexibility (E3). All respondents indicated that the intervention has the potential to be (at least partially) effective (E4), whereas no clear picture emerged regarding the cost-effectiveness of the intervention (E5). All therapists felt that the goals of the intervention match the prioritized goals (E6). 50% of the therapists agreed that the intervention can be piloted (E7), and all agreed that the intervention is reversible (E8). Transferring this intervention to clinical routine might be hindered by the need for specific training (B1) to deliver the intervention due to its complexity (B2). Furthermore, the intervention was rated as time-consuming (B3) and requiring ongoing support and supervision (B4). Some therapists believed that additional human resources (B5) would be necessary. No clear picture emerged regarding the need for additional material (B6) and the costs (B7). The majority did not think that the intervention had any SAEs for the patients (B8). The detailed SAFE evaluation is displayed in Fig. 2.

Among all patients, 135 AEs / SAEs were registered during the 28 days of intervention and TAU condition. Of these, 52% (69 AEs, 1 SAE) occurred in TAU and 48% in Mind it! (63 AEs, 2 SAEs). There were no differences between the groups regarding the number of (S)AEs.

## Discussion

The present study was among the first to apply a MBI to youth with SUD in an inpatient setting. As the intended sample size was not achieved, we provided both p-values and Cohen's effect sizes for interpretation. The main findings indicate a significant reduction in cannabis use days with a large effect size in both groups, demonstrating the success of inpatient treatment in reducing cannabis consumption. There were no significant group differences at post-treatment, but when considering the effect sizes for changes from pre to post, Mind it! appeared to be superior to TAU regarding reward and relief craving. Healthy self-regulation skills improved on both groups, a more pronounced effect for Mind it! becomes apparent later in the 6-month follow-up period. However, at the 6-month follow-up, TAU showed a significant reduction in SUD severity with a large effect size and reward craving, while Mind it! did not. Therapists found the intervention feasible. The following aspects will be discussed in light of the present results: (1) impact of our MBI on SUD-related behaviors, (2) feasibility of the adapted mindfulness treatment program, and (3) recruitment challenges for minors with SUD diagnoses in inpatient settings.

### Impact of Mind it! on SUD-related behaviors

Mind it! successfully improved healthy self-regulation, in line with previous work by Himelstein et al. [25], which served as the basis for our manual adaptation. Notably, our inpatient study revealed a reduction in cannabis use days, potentially due to the unique impact of the inpatient setting, in contrast to the outpatient studies by Himelstein et al. [25] and Harris et al. [26]. Regarding the change in craving as a direct effect of the intervention at post-treatment, earlier research revealed an inverse relationship between craving and mindfulness [28], which corresponds to the present findings. The positive impact of Mind it! on relief craving at post-treatment may be attributed to its focus on accepting unpleasant experiences (e.g. through urge surfing), aligning with the principles of mindfulness. However, the unexpected inverse effect at the 6-month follow-up challenges this hypothesis, as TAU showed greater reductions in SUD severity and craving, without superior gains in mindfulness. In sum, the results are inconclusive and challenging to decipher. Limited information is available about participants’ adherence to mindfulness exercises and additional treatment, potentially contributing to the lack of significant findings in relief craving at follow-up. Strengthening participants’ continued practice post-treatment could help to maintain positive effects, as research indicates that outcomes are closely tied to practice frequency [45].

### Feasibility of the adapted mindfulness treatment program

Another important consideration is the feasibility of the group format. While preliminary analyses indicated good acceptance and commitment to the mindfulness concept among inpatient youth with SUD [31], the low attendance rate (6 out of 12 sessions on average) is concerning, and potentially limits the full impact of the program. In our study, we aimed to factor in ward processes when planning group sessions. However, some patients missed sessions due to conflicts like other pre-scheduled appointments, difficulty managing these additional appointments, or feeling unwell (e. g. headaches). Adolescents with SUDs often show fluctuating and ambivalent motivation for change and treatment [46] which may also affect their motivation for engaging in exercises and reduce adherence to the group. Maybe the effectiveness of the intervention can be enhanced by modifying the number of group sessions, integrating the time point of appointments better to ward processes and needs of the patients and providing additional encouragement for mindfulness practice such as a reward for performing practice.

### Recruitment challenges for minors with SUD diagnoses in inpatient settings

Despite having two experienced university hospitals as recruitment centers, recruitment faced several challenges: (1) a significant proportion opted not to continue treatment after phase 1—which could also be attributed to the significant ambivalence towards treatment commonly observed in adolescents with SUD [46]. Recent meta-analytic evidence confirms dropout rates from inpatient SUD treatment about 30% in adult samples); however, greater levels of mental distress correlate with an elevated risk of dropout [47]. Given the severe illness of the current sample, this could explain the high rate of treatment discontinuation. (2) About one fifth of patients were excluded due to comorbid disorders like PTSD with flashbacks. This choice was informed by research indicating that mindfulness-based techniques can induce stress in clients vulnerable to flashbacks, potentially intensifying trauma memories [48, 49]. A subset of patients with comorbid PTSD diagnoses took part in the Mind it! group, as they did not report on being prone to flashbacks. Nonetheless, in some cases discontinuation of Mind it! had occurred among them. Future research should rigorously assess this patient subgroup, given the high prevalence of PTSD among adolescents with SUD (about 28%) and the negative association between traumatic symptoms and SUD severity [50]. 3) A notable proportion could not be reached for follow-up assessments. The study achieved an overall retention rate of 72.6%, slightly lower than in similar studies with adolescent SUD patients (e.g., [51]: 78.4%, [52]: 77.3%), potentially due to limited parent or guardian involvement in assessments and the complex backgrounds of participating patients with high comorbidity. Subsequent research must involve more than two centers to attain a sufficient sample size. However, the ambivalent treatment motivation seen in this patient group, along with frequent high drop-out rates, poses a challenge to implementing such group programs. To address this issue, it is essential to gain a deeper understanding of which individuals benefit from group therapy and are thus willing to endure the associated higher costs, as well as to enhance motivation for psychiatric treatment engagement. Introducing supplementary therapy modules to boost treatment motivation may be necessary as early as phase 1 of treatment.

## Strengths and limitations

This groundbreaking study is the first to rigorously examine a MBI within a multicenter, randomized controlled trial setting specifically designed for adolescent inpatients with SUD. Moreover, it offers a comprehensive follow-up period of 6 months. However, the study fell short of its intended sample size, with only 84 participants compared to the planned 246. Given the small sample size and limited group sessions, further research with more participants and more regular group participation is needed to validate potential effects. The interpretation of follow-up results is hampered by selective dropout among the intervention group, and we cannot ascertain whether group differences are due to the intervention or systematic difficulties in reaching participants for follow-up assessments. Patient satisfaction with Mind it! did not suggest dissatisfaction [31] as the cause for skewed response rates. Unfortunately, dropout analyses did not provide insights into the reasons for systematic dropout. However, the Mind it! group appeared to be more severely ill, exhibiting higher numbers of comorbid diagnoses, such as anxiety disorders; also borderline personality disorders was present in about one fifth of the patients. These disorders tend to present more challenges in terms of adherence and may therefore account for the systematic dropout [53]. Additionally, the study faces limitations due to low internal consistency of the SDS scale for assessing SUD severity. Finally, as a deviation from the study protocol, adherence and competency ratings were not carried out, therefore, despite intensive supervision, we cannot ensure fidelity to the MBI rationale.

## Future implications

In general, both groups showed improvements in most examined SUD-related factors and a reduction in cannabis consumption (primary outcome) with large effect size. At a low level, Mind it! appears to yield more favorable effects immediately post-treatment, whereas the TAU group demonstrates superior performance on SUD-related variables in the longer run. Mindfulness enhancement in form of healthy self-regulation is evident only in the Mind it! group in the long run. In sum, the effects on secondary parameters are inconsistent and challenging to interpret. Future research should consider two potential avenues: (1) analyze and optimize the Mind it! program for inpatient settings by enhancing therapy session participation and encouraging regular practice to maximize program effectiveness; and (2) offer the program in an outpatient setting, such as an aftercare program, following the model of the original studies by Himelstein et al. [25], to maintain achieved effects. Further investigations are needed to identify patient subgroups that may benefit more from the program e.g. by taking comorbidity into account and to explore additional outcomes like quality of life and emotion regulation.

## Supplementary Information

Below is the link to the electronic supplementary material.

Supplementary file1 (DOCX 46 KB)

## Data Availability

Upon reasonable request, deidentified data will be made available from the corresponding author.

## Footnotes

1 Cronbach’s α = 0.59 in the present sample.

2 Cronbach’s α = 0.89 in the present sample.

3 Instead of calculating scale scores, the authors advise reporting frequencies of staff’s answers for each factor (as in van der Krieke et al. [42]).
