# Supplementary material for: Mind it! A mindfulness-based group psychotherapy for substance use disorders in adolescent inpatients
Source: Eur Child Adolesc Psychiatry. 2024 May 15;33(12):4205–17. doi: 10.1007/s00787-024-02465-z (PMC11618143; doi:10.1007/s00787-024-02465-z)
Supplement: Supplementary file 1 — Supplementary file1 (DOCX 46 KB) [file 787_2024_2465_MOESM1_ESM.docx]

**SI 1** Additional information on dropout analyses

To analyze dropout effects across a broad spectrum of possible confounders, as a first step, we analyzed associations between availability of data at t3 and the following participant characteristics: group allocation, gender, inpatient treatment duration, adverse events, disciplinary discharge, trial center, and baseline scores of the following variables: cannabis craving (CCS-7), emotion regulation (DERS), global assessment of functioning, mindfulness (trait mindfulness: MAAS-A; mindfulness: MTASA), impulsivity (UPPS), psychosocial functioning (SDQ total problem score), severity of dependence (SDS), number of days with substance use within the past 30 days (for alcohol, cannabis, other illicit substances, and all substances, respectively). Not all variables mentioned here were analyzed in the present paper, but were collected as part of the study; see the study protocol (Baldus, Mokros et al., 2018) for more detailed information on the measures used. Significant associations, i.e., a larger number of unavailable data at t3 emerged for participants of the intervention group (phi coefficient = -.24, p = .028), participants with lower mindfulness according to the mindfulness domains (MTASA) paying active attention (r = .27, p = .021) and being aware of observations (r = .32, p = .004), and those with fewer overall substance use days within the past 30 days (r = .22, p = .047). As a further step, in an exploratory approach, we used logistic regressions to predict data availability at t3 from variables that were previously significant. A full model (Table x, Model 1) with all four variables showed no single predictor with p < .05. When reducing the model further by omitting the predictor with the lowest p-value in Model 1 (MTASA active attention), this new model (Model 2) showed meaningful effects for group allocation (OR = 0.30, p = .044) and MTASA awareness observation (OR = 3.58, p = .011) and no meaningful association with number of overall substance use days within the past 30 days (OR = 1.07, p = .121). A further reduction did not result in models with better model fit.

**Table SI1** Logistic regressions predicting availability of data at t3

| Predicted outcome | Predictors | OR | 95% CI | p | Model parameters |
| --- | --- | --- | --- | --- | --- |
| *Model 1:* |  |  |  |  |  |
| Data available at t3 | Group allocation  Days with substance use  MTASA awareness observation  MTASA active attention | 0.33  1.08  2.51  1.87 | 0.10 – 1.06  0.98 – 1.17  0.77 – 8.21  0.48 – 7.28 | .063  .107  .127  .368 | χ^2^= 14.50, df = 4, p = .006  Nagelkerke’s R^2^ = 0.26 |
| *Model 2* |  |  |  |  |  |
| Data available at t3 | Group allocation  Days with substance use  MTASA awareness observation | 0.30  1.07  3.58 | 0.09 – 0.97  0.98 – 1.17  1.34 -9.55 | .044  .121  .011 | χ^2^= 14.72, df = 3, p = .002  Nagelkerke’s R^2^ = 0.25 |
|  |  |  |  |  |  |

Notes: Data available at t3 (0 = no; 1 = yes); Group allocation (1 = Mind it! group; 0 = Control group) , OR = Odds Ratio, CI = Confidence interval

**SI2 Procedure of data imputation for sensitivity analyses and results of the sensitivity analyses**

To identify variables for this imputation model, we calculated Spearman rank correlations between the relevant baseline characteristics and dropout (yes/no) and then performed a logistic regression analysis with dropout (yes vs. no) as outcome and those baseline variables that previously showed significant correlations (p < 0.05) as independent variables. In this variable selection approach, the p-value is used only in a descriptive manner. The following baseline characteristics were included in the model: gender, age, sociodemographic characteristics, craving, emotion regulation, quality of life, mindfulness aspects, stress, impulsivity, severity of dependence, substance use, and number of comorbid diagnoses. We created 20 imputed data sets, and the result of each run was combined using Rubin’s Rules to obtain a pooled result .

**Table SI2** Results of analyses using imputed data sets for use days for the assessed substances at baseline and follow-up assessment

|  |  | Change from baseline | | | | | | |  | Between-group differences (CG - IG) | | |  | Effect size |
| --- | --- | --- | --- | --- | --- | --- | --- | --- | --- | --- | --- | --- | --- | --- |
|  | Mind it! group | | | |  | Control group | | |  |  |  |  |  |  |
|  | EM | | 95% CI | d |  | EM | 95% CI | d |  | EM | 95% CI | p |  | d |
| **TLFB** |  | |  |  |  |  |  |  |  |  |  |  |  |  |
| Cannabis use days |  | |  |  |  |  |  |  |  |  |  |  |  |  |
| t0 to t3 | -8.74 | | -12.31 - -5.18 | -0.74 |  | -9.66 | -13.12 - -6.19 | -0.84 |  | -0.91 | -5.84 – 4.01 | .716 |  | 0.08 |
| Alcohol use days |  | |  |  |  |  |  |  |  |  |  |  |  |  |
| t0 to t3 | -0.35 | | -2.45 – 1.76 | -0.05 |  | -2.70 | -4.76 - -0.64 | -0.38 |  | -2.35 | -5.27 – 0.57 | .114 |  | 0.08 |
| Overall use days |  | |  |  |  |  |  |  |  |  |  |  |  |  |
| t0 to t3 | -1.12 | | -3.03 – 0.78 | -0.18 |  | -1.56 | -3.41 – 0.30 | -0.25 |  | -0.43 | -3.08 – 2.21 | .747 |  | 0.07 |

t0 = baseline, t3 = follow-up

**Note** EM=Estimated Marginal Means for differences, negative target values represent a decrease in substance use between T0 and T3, positive values represent an increase in use. Effect size for intervention – A negative score indicated a decrease in number of use days within the past 30 days between baseline and follow-up, a negative effect size (Cohen’s d) is associated with a favorable intervention effect in the Mind it! group. CI=95% confidence interval; d: Cohen’s d; CG: control group; IG: Mind it! intervention group; T0: Baseline; T3: follow-up. TLFB = Timeline Followback interview (last 30 days prior to interview)

**Table SI3** Imputed data – SUD-related outcomes

|  |  | Change from baseline | | | | | | |  | Between-group differences (IG - CG) | | |  | Effect size |
| --- | --- | --- | --- | --- | --- | --- | --- | --- | --- | --- | --- | --- | --- | --- |
|  | Mind it! Intervention group | | | |  | Control group | | |  |  |  |  |  |  |
|  | EM | | 95% CI | d^#^ |  | EM | 95% CI | d^#^ |  | EM | 95% CI | p |  | d^*^ |
| **SDS** |  | |  |  |  |  |  |  |  |  |  |  |  |  |
| T0 to T2 | -0.03 | | -0.80 - 0.74 | -0.01 |  | 0.26 | -0.49 - 0.94 | 0.11 |  | -0.29 | -1.29 – 0.71 | .566 |  | -0.12 |
| T0 to T3 | 0.16 | | -0.64 - 0.97 | 0.06 |  | -1.05 | -1.81- -0.29 | -0.42 |  | 1.21 | 0.22 – 2.21 | .018 |  | 0.47 |
| **CCS7** |  | |  |  |  |  |  |  |  |  |  |  |  |  |
| Reward craving |  | |  |  |  |  |  |  |  |  |  |  |  |  |
| T0 to T2 | -0.54 | | -1.09 - 0.02 | -0.29 |  | -0.18 | -0.73 - 0.37 | -0.10 |  | -0.20 | -0.97 – 0.57 | .603 |  | -0.20 |
| T0 to T3 | -0.07 | | -0.68 - 0.55 | -0.03 |  | -0.44 | -1.02 - 0.14 | -0.23 |  | 0.49 | -0.28 – 1.25 | .215 |  | 0.19 |
| Relief craving |  | |  |  |  |  |  |  |  |  |  |  |  |  |
| T0 to T2 | -0.84 | | -1.29 - -0.39 | -0.57 |  | -0.12 | -0.57 - 0.33 | -0.08 |  | -0.69 | -1.24 - -0.13 | 0.156 |  | -0.49 |
| T0 to T3 | -0.42 | | -0.87 - 0.03 | -0.28 |  | -0.52 | -0.98 - -0.06 | -0.35 |  | 0.07 | -0.49 – 0.62 | .814 |  | 0.07 |

**Note** EM=Estimated Marginal Means for differences, CI=95% confidence interval; effect size d: Cohen’s d; CG: control group; IG: Mind it! intervention group; t0: Baseline; t2: post-assessment; t3: follow-up. SDS =Severity of Dependence Scale; CCS-7 = Cannabis Craving Screening short form,

^#^ negative d-score associated with favorable development over time

^*^ negative d score associated with better results for Mind it! group vs. control group

**Table SI4** Results of analyses using multiple imputation data sets for mindfulness-related outcomes

|  |  | Change from baseline | | | | | | |  | Between-group differences (IG - CG)^7^ | | |  | Effect size |
| --- | --- | --- | --- | --- | --- | --- | --- | --- | --- | --- | --- | --- | --- | --- |
|  | Mind it! group | | | |  | Control group | | |  |  |  |  |  |  |
|  | Adjusted Mean | | 95% CI | d^#^ |  | Adjusted Mean | 95% CI | d^#^ |  | Adjusted Mean | 95% CI | p |  | d^*^ |
| **MTASA** |  | |  |  |  |  |  |  |  |  |  |  |  |  |
| Healthy self-regulation |  | |  |  |  |  |  |  |  |  |  |  |  |  |
| t0 to t2 | 0.21 | | -0.02 - 0.44 | 0.27 |  | 0.11 | -0.12 - 0.33 | 0.14 |  | 0.10 | -0.17 – 0.37 | .451 |  | 0.14 |
| t0 to t3 | 0.28 | | 0.03 - 0.52 | 0.35 |  | 0.11 | -0.12 - 0.34 | 0.14 |  | 0.17 | -0.10 – 0.44 | .210 |  | 0.22 |
| **MAAS-A** |  | |  |  |  |  |  |  |  |  |  |  |  |  |
|  |  | |  |  |  |  |  |  |  |  |  |  |  |  |
| t0 to t2 | 3.39 | | 0.23 - 6.55 | 0.32 |  | 3.46 | 0.33 - 6.58 | 0.33 |  | -0.07 | -4.46 - 4.33 | .976 |  | -0.01 |
| t0 to t3 | 0.59 | | -2.66 - 3.84 | 0.06 |  | 1.95 | -1.20 - 5.10 | 0.19 |  | -1.35 | -5.75 - 3.04 | .543 |  | -0.13 |

t0 = baseline, t1 = interim, t2 = post, t3 = follow-up

**Note** EM=Estimated Marginal Means for differences, CI=95% confidence interval; effect size d: Cohen’s d; CG: control group; IG: Mind It! intervention group; t0: Baseline; t2: post-assessment; t3: follow-up. MTASA = Mindful Thinking and Action Scale for Adolescents; MAAS-A: Mindful Attention Awareness Scale for Adolescents.

^#^ positive d-score indicates better healthy self-regulation development over time

^*^ positive d-score associated with better results for Mind it! than control group

**Table SI5**

*Overview of the content of the eight Core Sessions and the four Booster Sessions*

| Core Sessions  (2x per week) | Topic and content of each session |
| --- | --- |
| *Session 1* | - Introduction: The concept of mindfulness - Mindfulness of the breath |
| *Session 2* | - Difference between (unconsidered, automated) reaction vs. responding |
| *Session 3* | - Mindfulness-based decision-making technique: SAVE (stop, breathe, imagine the consequences, decide) - Mindfulness oft the breath |
| *Session 4* | - Substance-Abuse education - Pleasant, short-term feelings of intoxication through substance use vs. lasting feelings of happiness through turning inward - Bodyscan |
| *Session 5* | - Emotions and their different qualities of experience against the background of different life experiences - „Stand if“ exercise |
| *Session 6* | - Universality of unpleasant feelings - Exercise for self-revelation of a significant, personal event or alternatively: "Tree of Emotions" exercise - Self-Compassion meditation - De-Escalation exercise |
| *Session 7* | - Dealing with cravings in the case of substance abuse - Non-reactive coping with craving („urge-surfing“) - Nonmoving body scan (non-reactivity) |
| *Session 8* | - Repetition: Dealing with craving - Individual causes of the participants' cravings - Nonmoving bodyscan |
| Booster Session  (1x weekly) |  |
| *Session 1* | - The family system and drugs - Mindfulness as a support for building or restoring family cohesion - Meditation exercises |
| *Session 2* | - Relationship skills and setting boundaries with peers |
| *Session 3* | - Repetition of the "SAVE" technique - Walking meditation (mindful experience of everyday moments) |
| *Session 4* | - Discuss the exercise "mindful everyday moments" - Repetition the definitions of formal vs. informal mindfulness - Formal mindfulness exercise: mindful eating of fizzy sweets |

| Domain | Original study protocol | Implemented change | Rational/comments |
| --- | --- | --- | --- |
|  |  |  |  |
| Design | confirmatory randomized controlled trial with n=246 participants | pilot randomized controlled study focusing on the feasibility of Mind it! with n= 84 participants | due to recruitment and adherence challenges (see also [25]) |
| Statistical Analysis | Mixed negative binomial regression | Linear regression | Omission of random effects:  Substance use was only measured once after the intervention, so it makes no sense to want to correct for repeated measurements at this point. The therapy groups were designed as open groups from the outset, so it is not possible to model a cluster effect. This would have been possible with a closed form.  After publication of the study protocol, it was determined that this type of data can be modeled well with a linear model as a change from baseline. The requirements (normality of residuals) were met and the results are easier to interpret. In this respect, the decision to change the primary analysis was made before the start of any analysis. |
| secondary outcomes | Large number of secondary outcomes | Limited number of secondary outcomes | To sharpen the focus and due to character limitation. The reported outcomes were selected prior to analysis. |

**Table SI6: Changes in study protocol**
